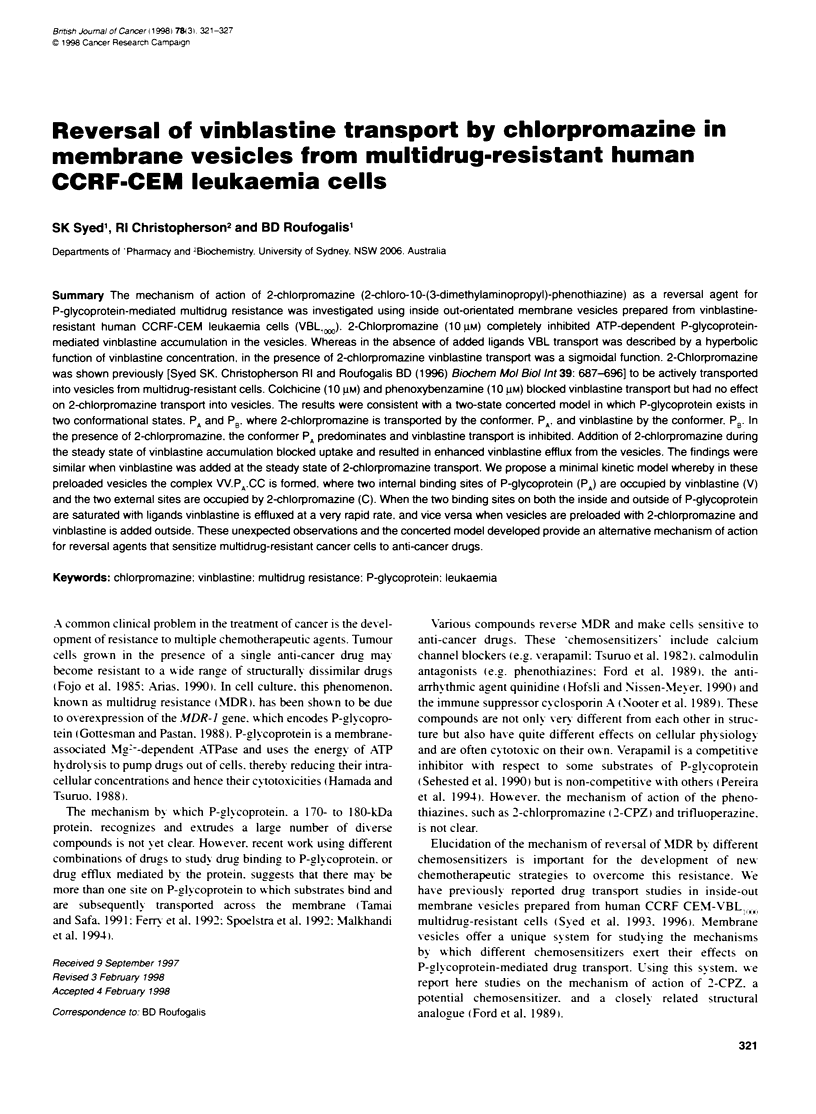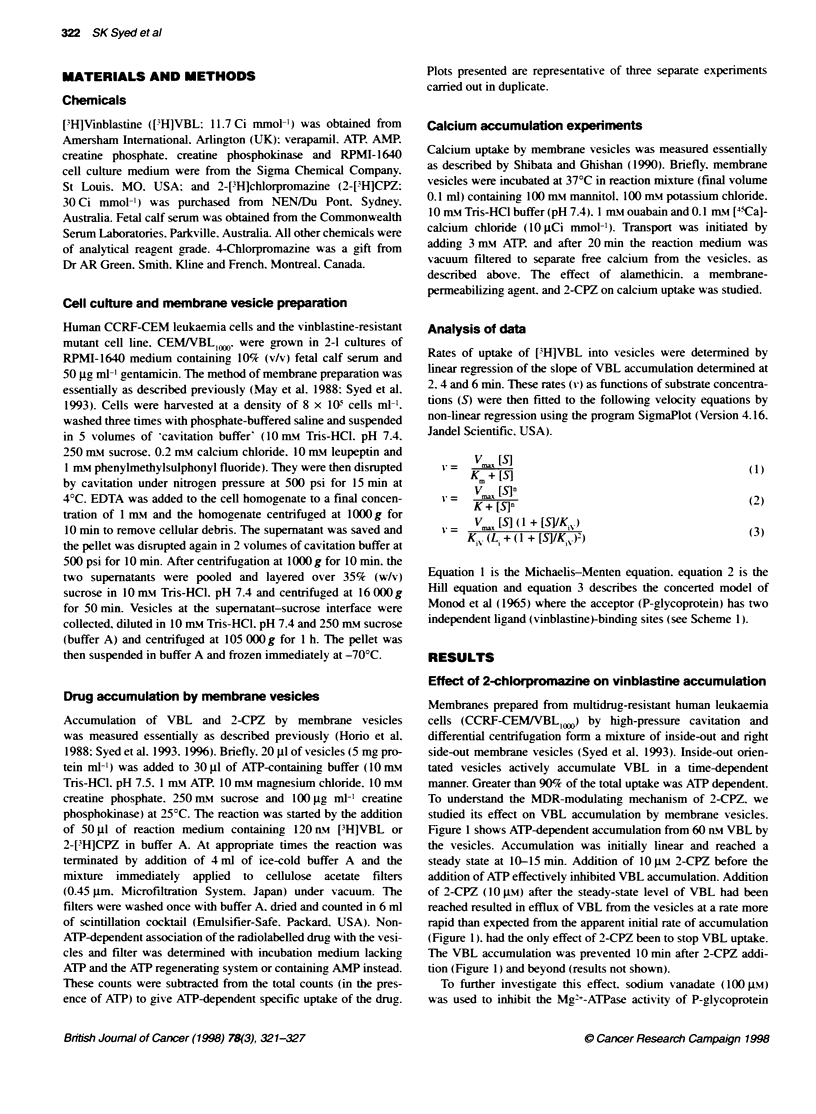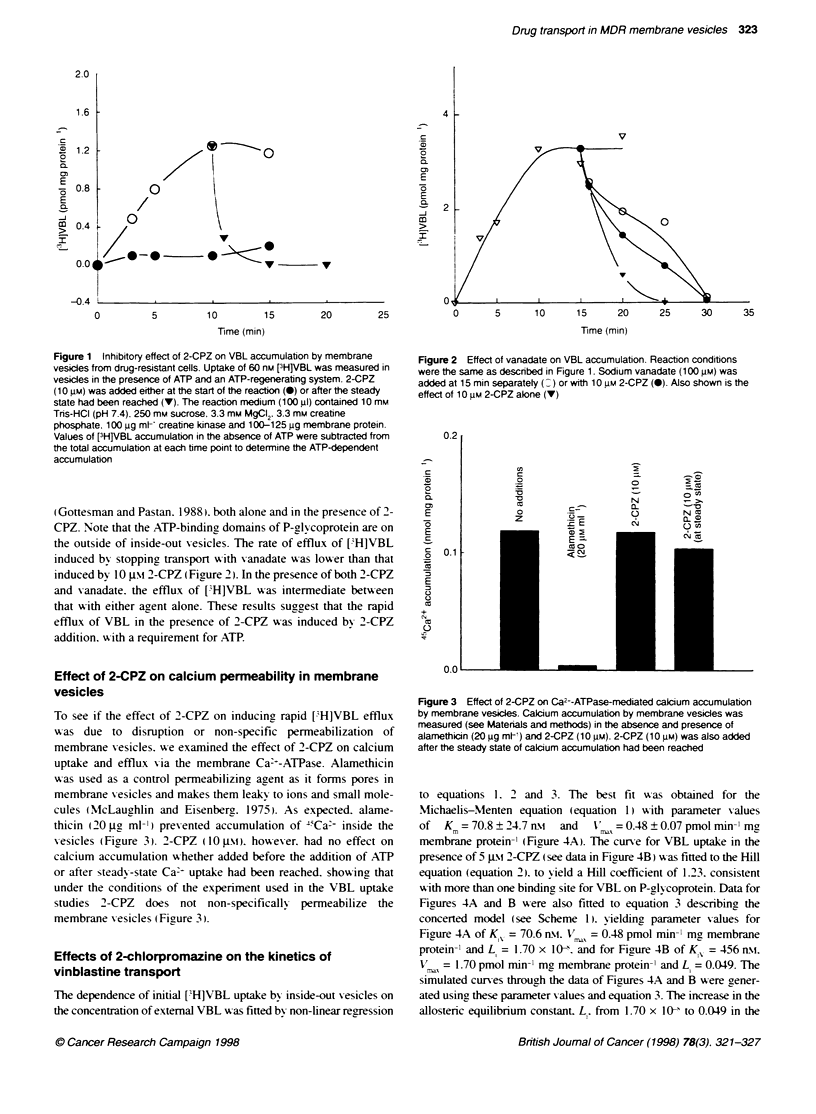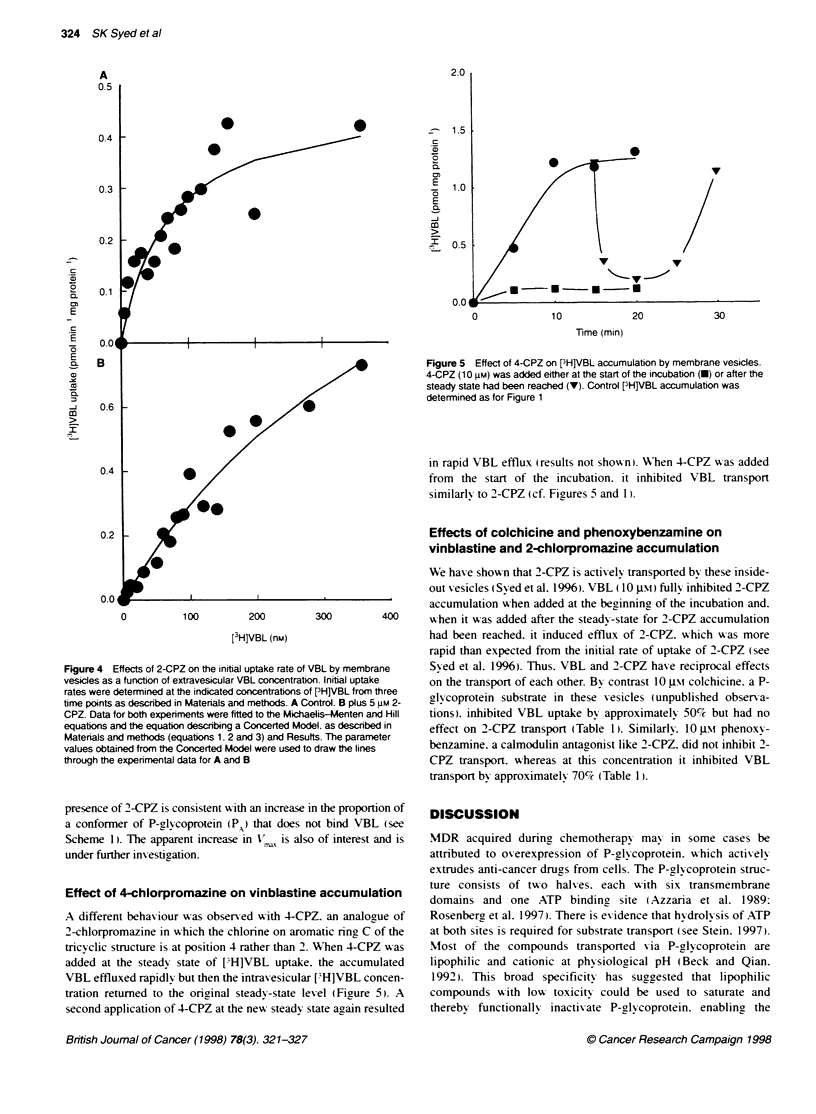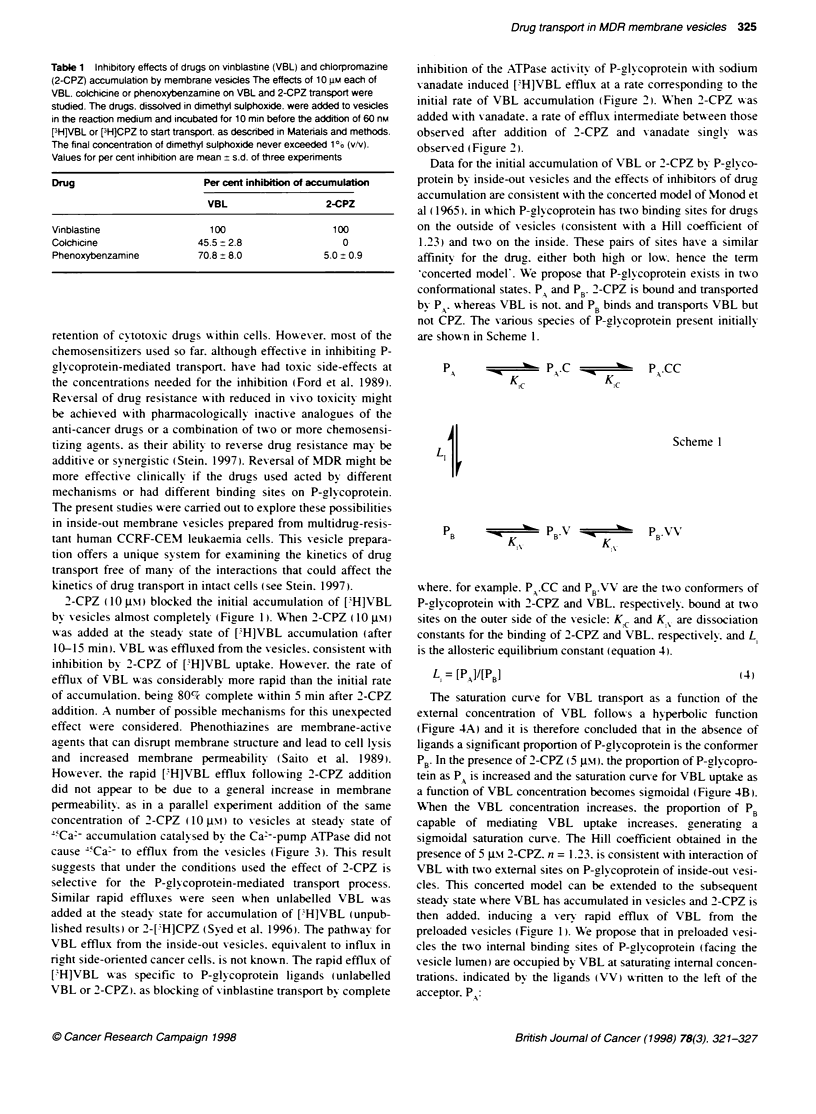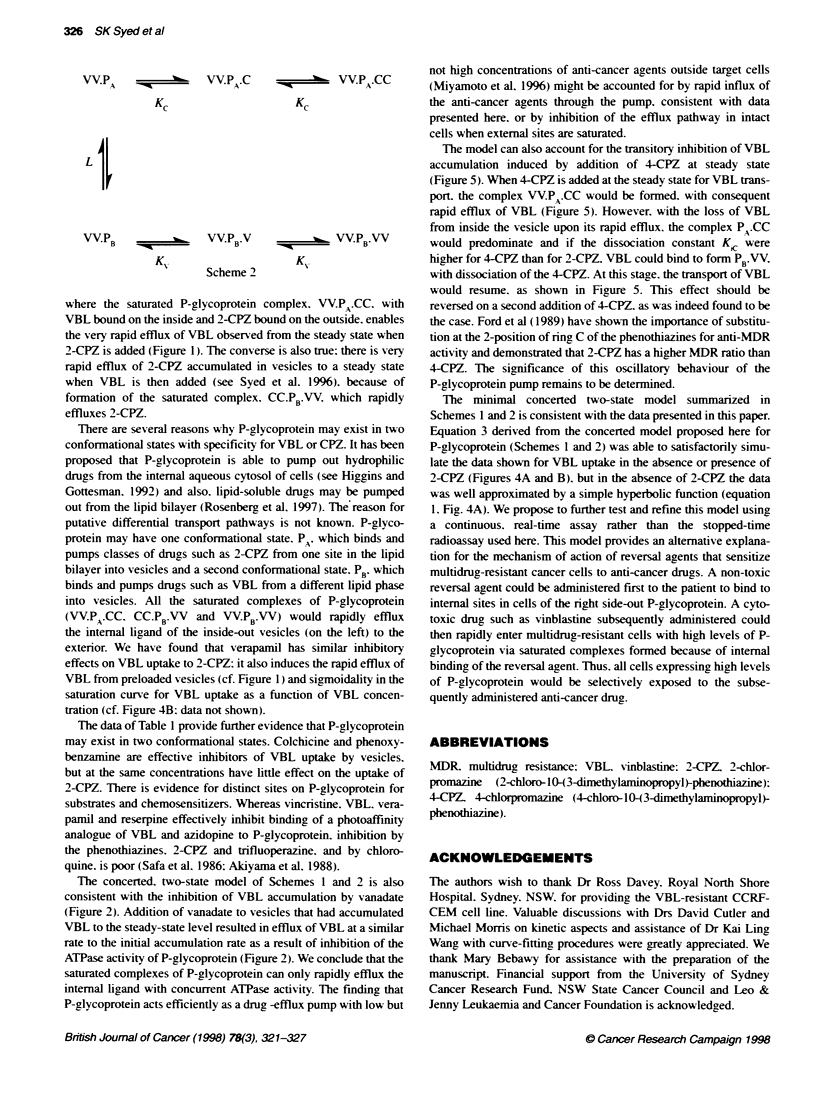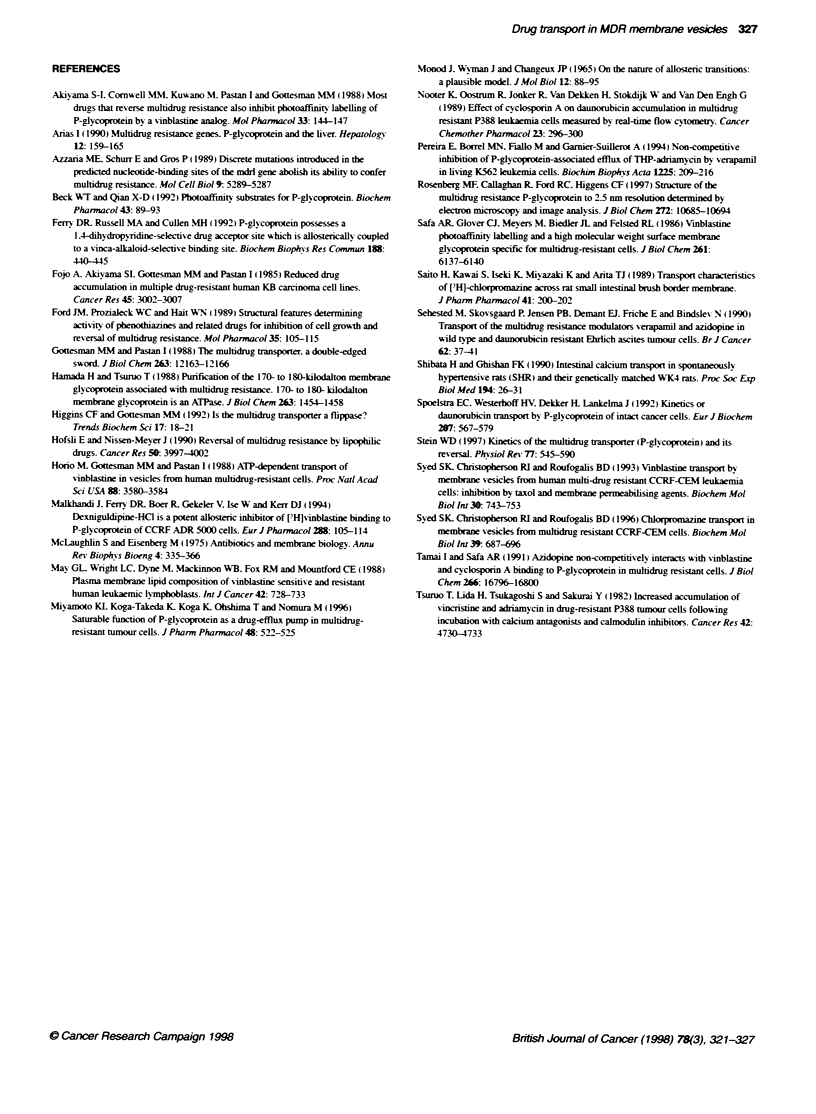# Reversal of vinblastine transport by chlorpromazine in membrane vesicles from multidrug-resistant human CCRF-CEM leukaemia cells.

**DOI:** 10.1038/bjc.1998.493

**Published:** 1998-08

**Authors:** S. K. Syed, R. I. Christopherson, B. D. Roufogalis

**Affiliations:** Department of Pharmacy, University of Sydney, NSW, Australia.

## Abstract

The mechanism of action of 2-chlorpromazine (2-chloro-10-(3-dimethylaminopropyl)-phenothiazine) as a reversal agent for P-glycoprotein-mediated multidrug resistance was investigated using inside out-orientated membrane vesicles prepared from vinblastine-resistant human CCRF-CEM leukaemia cells (VBL1000). 2-Chlorpromazine (10 microM) completely inhibited ATP-dependent P-glycoprotein-mediated vinblastine accumulation in the vesicles. Whereas in the absence of added ligands VBL transport was described by a hyperbolic function of vinblastine concentration, in the presence of 2-chlorpromazine vinblastine transport was a sigmoidal function. 2-Chlorpromazine was shown previously [Syed SK, Christopherson RI and Roufogalis BD (1996) Biochem Mol Biol Int 39: 687-696] to be actively transported into vesicles from multidrug-resistant cells. Colchicine (10 microM) and phenoxybenzamine (10 microM) blocked vinblastine transport but had no effect on 2-chlorpromazine transport into vesicles. The results were consistent with a two-state concerted model in which P-glycoprotein exists in two conformational states, P(A) and P(B), where 2-chlorpromazine is transported by the conformer, P(A), and vinblastine by the conformer, P(B). In the presence of 2-chlorpromazine, the conformer P(A) predominates and vinblastine transport is inhibited. Addition of 2-chlorpromazine during the steady state of vinblastine accumulation blocked uptake and resulted in enhanced vinblastine efflux from the vesicles. The findings were similar when vinblastine was added at the steady state of 2-chlorpromazine transport. We propose a minimal kinetic model whereby in these preloaded vesicles the complex VV.P(A).CC is formed, where two internal binding sites of P-glycoprotein (P(A)) are occupied by vinblastine (V) and the two external sites are occupied by 2-chlorpromazine (C). When the two binding sites on both the inside and outside of P-glycoprotein are saturated with ligands vinblastine is effluxed at a very rapid rate, and vice versa when vesicles are preloaded with 2-chlorpromazine and vinblastine is added outside. These unexpected observations and the concerted model developed provide an alternative mechanism of action for reversal agents that sensitize multidrug-resistant cancer cells to anti-cancer drugs.


British Journal of Cancer (1998) 78(3), 321–327
© 1998 Cancer Research Campaign

### Reversal of vinblastine transport by chlorpromazine in membrane vesicles from multidrug-resistant human CCRF-CEM leukaemia cells

**SK Syed[1], RI Christopherson[2] and BD Roufogalis[1]**

Departments of [1]Pharmacy and [2]Biochemistry, University of Sydney, NSW 2006, Australia

**Summary** The mechanism of action of 2-chlorpromazine (2-chloro-10-(3-dimethylaminopropyl)-phenothiazine) as a reversal agent for P-glycoprotein-mediated multidrug resistance was investigated using inside out-orientated membrane vesicles prepared from vinblastine-resistant human CCRF-CEM leukaemia cells ($VBL_{1000}$). 2-Chlorpromazine (10 μM) completely inhibited ATP-dependent P-glycoprotein-mediated vinblastine accumulation in the vesicles. Whereas in the absence of added ligands VBL transport was described by a hyperbolic function of vinblastine concentration, in the presence of 2-chlorpromazine vinblastine transport was a sigmoidal function. 2-Chlorpromazine was shown previously [Syed SK, Christopherson RI and Roufogalis BD (1996) *Biochem Mol Biol Int* **39**: 687–696] to be actively transported into vesicles from multidrug-resistant cells. Colchicine (10 μM) and phenoxybenzamine (10 μM) blocked vinblastine transport but had no effect on 2-chlorpromazine transport into vesicles. The results were consistent with a two-state concerted model in which P-glycoprotein exists in two conformational states, $P_A$ and $P_B$, where 2-chlorpromazine is transported by the conformer, $P_A$, and vinblastine by the conformer, $P_B$. In the presence of 2-chlorpromazine, the conformer $P_A$ predominates and vinblastine transport is inhibited. Addition of 2-chlorpromazine during the steady state of vinblastine accumulation blocked uptake and resulted in enhanced vinblastine efflux from the vesicles. The findings were similar when vinblastine was added at the steady state of 2-chlorpromazine transport. We propose a minimal kinetic model whereby in these preloaded vesicles the complex $VV.P_A.CC$ is formed, where two internal binding sites of P-glycoprotein ($P_A$) are occupied by vinblastine (V) and the two external sites are occupied by 2-chlorpromazine (C). When the two binding sites on both the inside and outside of P-glycoprotein are saturated with ligands vinblastine is effluxed at a very rapid rate, and vice versa when vesicles are preloaded with 2-chlorpromazine and vinblastine is added outside. These unexpected observations and the concerted model developed provide an alternative mechanism of action for reversal agents that sensitize multidrug-resistant cancer cells to anti-cancer drugs.

**Keywords:** chlorpromazine; vinblastine; multidrug resistance; P-glycoprotein; leukaemia

A common clinical problem in the treatment of cancer is the development of resistance to multiple chemotherapeutic agents. Tumour cells grown in the presence of a single anti-cancer drug may become resistant to a wide range of structurally dissimilar drugs (Fojo et al, 1985; Arias, 1990). In cell culture, this phenomenon, known as multidrug resistance (MDR), has been shown to be due to overexpression of the *MDR-1* gene, which encodes P-glycoprotein (Gottesman and Pastan, 1988). P-glycoprotein is a membrane-associated $Mg^{2+}$-dependent ATPase and uses the energy of ATP hydrolysis to pump drugs out of cells, thereby reducing their intracellular concentrations and hence their cytotoxicities (Hamada and Tsuruo, 1988).

The mechanism by which P-glycoprotein, a 170- to 180-kDa protein, recognizes and extrudes a large number of diverse compounds is not yet clear. However, recent work using different combinations of drugs to study drug binding to P-glycoprotein, or drug efflux mediated by the protein, suggests that there may be more than one site on P-glycoprotein to which substrates bind and are subsequently transported across the membrane (Tamai and Safa, 1991; Ferry et al, 1992; Spoelstra et al, 1992; Malkhandi et al, 1994).

*Received 9 September 1997*
*Revised 3 February 1998*
*Accepted 4 February 1998*

*Correspondence to:* BD Roufogalis

Various compounds reverse MDR and make cells sensitive to anti-cancer drugs. These 'chemosensitizers' include calcium channel blockers (e.g. verapamil; Tsuruo et al, 1982), calmodulin antagonists (e.g. phenothiazines; Ford et al, 1989), the anti-arrhythmic agent quinidine (Hofsli and Nissen-Meyer, 1990) and the immune suppressor cyclosporin A (Nooter et al, 1989). These compounds are not only very different from each other in structure but also have quite different effects on cellular physiology and are often cytotoxic on their own. Verapamil is a competitive inhibitor with respect to some substrates of P-glycoprotein (Sehested et al, 1990) but is non-competitive with others (Pereira et al, 1994). However, the mechanism of action of the phenothiazines, such as 2-chlorpromazine (2-CPZ) and trifluoperazine, is not clear.

Elucidation of the mechanism of reversal of MDR by different chemosensitizers is important for the development of new chemotherapeutic strategies to overcome this resistance. We have previously reported drug transport studies in inside-out membrane vesicles prepared from human CCRF CEM-$VBL_{1000}$ multidrug-resistant cells (Syed et al, 1993, 1996). Membrane vesicles offer a unique system for studying the mechanisms by which different chemosensitizers exert their effects on P-glycoprotein-mediated drug transport. Using this system, we report here studies on the mechanism of action of 2-CPZ, a potential chemosensitizer, and a closely related structural analogue (Ford et al, 1989).

### MATERIALS AND METHODS

### Chemicals

[$^3$H]Vinblastine ([$^3$H]VBL; 11.7 Ci mmol$^{-1}$) was obtained from Amersham International, Arlington (UK); verapamil, ATP, AMP, creatine phosphate, creatine phosphokinase and RPMI-1640 cell culture medium were from the Sigma Chemical Company, St Louis, MO, USA; and 2-[$^3$H]chlorpromazine (2-[$^3$H]CPZ; 30 Ci mmol$^{-1}$) was purchased from NEN/Du Pont, Sydney, Australia. Fetal calf serum was obtained from the Commonwealth Serum Laboratories, Parkville, Australia. All other chemicals were of analytical reagent grade. 4-Chlorpromazine was a gift from Dr AR Green, Smith, Kline and French, Montreal, Canada.

### Cell culture and membrane vesicle preparation

Human CCRF-CEM leukaemia cells and the vinblastine-resistant mutant cell line, CEM/VBL$_{1000}$, were grown in 2-l cultures of RPMI-1640 medium containing 10% (v/v) fetal calf serum and 50 µg ml$^{-1}$ gentamicin. The method of membrane preparation was essentially as described previously (May et al, 1988; Syed et al, 1993). Cells were harvested at a density of 8 × 10$^5$ cells ml$^{-1}$, washed three times with phosphate-buffered saline and suspended in 5 volumes of 'cavitation buffer' (10 mM Tris-HCl, pH 7.4, 250 mM sucrose, 0.2 mM calcium chloride, 10 mM leupeptin and 1 mM phenylmethylsulphonyl fluoride). They were then disrupted by cavitation under nitrogen pressure at 500 psi for 15 min at 4°C. EDTA was added to the cell homogenate to a final concentration of 1 mM and the homogenate centrifuged at 1000 ***g*** for 10 min to remove cellular debris. The supernatant was saved and the pellet was disrupted again in 2 volumes of cavitation buffer at 500 psi for 10 min. After centrifugation at 1000 ***g*** for 10 min, the two supernatants were pooled and layered over 35% (w/v) sucrose in 10 mM Tris-HCl, pH 7.4 and centrifuged at 16 000 ***g*** for 50 min. Vesicles at the supernatant–sucrose interface were collected, diluted in 10 mM Tris-HCl, pH 7.4 and 250 mM sucrose (buffer A) and centrifuged at 105 000 ***g*** for 1 h. The pellet was then suspended in buffer A and frozen immediately at –70°C.

### Drug accumulation by membrane vesicles

Accumulation of VBL and 2-CPZ by membrane vesicles was measured essentially as described previously (Horio et al, 1988; Syed et al, 1993, 1996). Briefly, 20 µl of vesicles (5 mg protein ml$^{-1}$) was added to 30 µl of ATP-containing buffer (10 mM Tris-HCl, pH 7.5, 1 mM ATP, 10 mM magnesium chloride, 10 mM creatine phosphate, 250 mM sucrose and 100 µg ml$^{-1}$ creatine phosphokinase) at 25°C. The reaction was started by the addition of 50 µl of reaction medium containing 120 nM [$^3$H]VBL or 2-[$^3$H]CPZ in buffer A. At appropriate times the reaction was terminated by addition of 4 ml of ice-cold buffer A and the mixture immediately applied to cellulose acetate filters (0.45 µm, Microfiltration System, Japan) under vacuum. The filters were washed once with buffer A, dried and counted in 6 ml of scintillation cocktail (Emulsifier-Safe, Packard, USA). Non-ATP-dependent association of the radiolabelled drug with the vesicles and filter was determined with incubation medium lacking ATP and the ATP regenerating system or containing AMP instead. These counts were subtracted from the total counts (in the presence of ATP) to give ATP-dependent specific uptake of the drug. Plots presented are representative of three separate experiments carried out in duplicate.

### Calcium accumulation experiments

Calcium uptake by membrane vesicles was measured essentially as described by Shibata and Ghishan (1990). Briefly, membrane vesicles were incubated at 37°C in reaction mixture (final volume 0.1 ml) containing 100 mM mannitol, 100 mM potassium chloride, 10 mM Tris-HCl buffer (pH 7.4), 1 mM ouabain and 0.1 mM [$^{45}$Ca]-calcium chloride (10 µCi mmol$^{-1}$). Transport was initiated by adding 3 mM ATP, and after 20 min the reaction medium was vacuum filtered to separate free calcium from the vesicles, as described above. The effect of alamethicin, a membrane-permeabilizing agent, and 2-CPZ on calcium uptake was studied.

### Analysis of data

Rates of uptake of [$^3$H]VBL into vesicles were determined by linear regression of the slope of VBL accumulation determined at 2, 4 and 6 min. These rates ($v$) as functions of substrate concentrations ($S$) were then fitted to the following velocity equations by non-linear regression using the program SigmaPlot (Version 4.16, Jandel Scientific, USA).

$$v = \frac{V_{max}[S]}{K_m + [S]} \quad (1)$$

$$v = \frac{V_{max}[S]^n}{K + [S]^n} \quad (2)$$

$$v = \frac{V_{max}[S](1 + [S]/K_{iV})}{K_{iV}(L_i + (1 + [S]/K_{iV})^2)} \quad (3)$$

Equation 1 is the Michaelis–Menten equation, equation 2 is the Hill equation and equation 3 describes the concerted model of Monod et al (1965) where the acceptor (P-glycoprotein) has two independent ligand (vinblastine)-binding sites (see Scheme 1).

### RESULTS

### Effect of 2-chlorpromazine on vinblastine accumulation

Membranes prepared from multidrug-resistant human leukaemia cells (CCRF-CEM/VBL$_{1000}$) by high-pressure cavitation and differential centrifugation form a mixture of inside-out and right side-out membrane vesicles (Syed et al, 1993). Inside-out orientated vesicles actively accumulate VBL in a time-dependent manner. Greater than 90% of the total uptake was ATP dependent. To understand the MDR-modulating mechanism of 2-CPZ, we studied its effect on VBL accumulation by membrane vesicles. Figure 1 shows ATP-dependent accumulation from 60 nM VBL by the vesicles. Accumulation was initially linear and reached a steady state at 10–15 min. Addition of 10 µM 2-CPZ before the addition of ATP effectively inhibited VBL accumulation. Addition of 2-CPZ (10 µM) after the steady-state level of VBL had been reached resulted in efflux of VBL from the vesicles at a rate more rapid than expected from the apparent initial rate of accumulation (Figure 1), had the only effect of 2-CPZ been to stop VBL uptake. The VBL accumulation was prevented 10 min after 2-CPZ addition (Figure 1) and beyond (results not shown).

To further investigate this effect, sodium vanadate (100 µM) was used to inhibit the $Mg^{2+}$-ATPase activity of P-glycoprotein

© Cancer Research Campaign 1998

**Figure 1** Inhibitory effect of 2-CPZ on VBL accumulation by membrane vesicles from drug-resistant cells. Uptake of 60 nM $[^3H]$VBL was measured in vesicles in the presence of ATP and an ATP-regenerating system. 2-CPZ (10 μM) was added either at the start of the reaction (●) or after the steady state had been reached (▼). The reaction medium (100 μl) contained 10 mM Tris-HCl (pH 7.4), 250 mM sucrose, 3.3 mM $MgCl_2$, 3.3 mM creatine phosphate, 100 μg ml⁻¹ creatine kinase and 100–125 μg membrane protein. Values of $[^3H]$VBL accumulation in the absence of ATP were subtracted from the total accumulation at each time point to determine the ATP-dependent accumulation

**Figure 2** Effect of vanadate on VBL accumulation. Reaction conditions were the same as described in Figure 1. Sodium vanadate (100 μM) was added at 15 min separately (○) or with 10 μM 2-CPZ (●). Also shown is the effect of 10 μM 2-CPZ alone (▼)

(Gottesman and Pastan, 1988), both alone and in the presence of 2-CPZ. Note that the ATP-binding domains of P-glycoprotein are on the outside of inside-out vesicles. The rate of efflux of $[^3H]$VBL induced by stopping transport with vanadate was lower than that induced by 10 μM 2-CPZ (Figure 2). In the presence of both 2-CPZ and vanadate, the efflux of $[^3H]$VBL was intermediate between that with either agent alone. These results suggest that the rapid efflux of VBL in the presence of 2-CPZ was induced by 2-CPZ addition, with a requirement for ATP.

### Effect of 2-CPZ on calcium permeability in membrane vesicles

To see if the effect of 2-CPZ on inducing rapid $[^3H]$VBL efflux was due to disruption or non-specific permeabilization of membrane vesicles, we examined the effect of 2-CPZ on calcium uptake and efflux via the membrane $Ca^{2+}$-ATPase. Alamethicin was used as a control permeabilizing agent as it forms pores in membrane vesicles and makes them leaky to ions and small molecules (McLaughlin and Eisenberg, 1975). As expected, alamethicin (20 μg ml⁻¹) prevented accumulation of $^{45}Ca^{2+}$ inside the vesicles (Figure 3). 2-CPZ (10 μM), however, had no effect on calcium accumulation whether added before the addition of ATP or after steady-state $Ca^{2+}$ uptake had been reached, showing that under the conditions of the experiment used in the VBL uptake studies 2-CPZ does not non-specifically permeabilize the membrane vesicles (Figure 3).

### Effects of 2-chlorpromazine on the kinetics of vinblastine transport

The dependence of initial $[^3H]$VBL uptake by inside-out vesicles on the concentration of external VBL was fitted by non-linear regression

**Figure 3** Effect of 2-CPZ on $Ca^{2+}$-ATPase-mediated calcium accumulation by membrane vesicles. Calcium accumulation by membrane vesicles was measured (see Materials and methods) in the absence and presence of alamethicin (20 μg ml⁻¹) and 2-CPZ (10 μM). 2-CPZ (10 μM) was also added after the steady state of calcium accumulation had been reached to equations 1, 2 and 3. The best fit was obtained for the Michaelis–Menten equation (equation 1) with parameter values of $K_m = 70.8 \pm 24.7$ nM and $V_{max} = 0.48 \pm 0.07$ pmol min⁻¹ mg membrane protein⁻¹ (Figure 4A). The curve for VBL uptake in the presence of 5 μM 2-CPZ (see data in Figure 4B) was fitted to the Hill equation (equation 2), to yield a Hill coefficient of 1.23, consistent with more than one binding site for VBL on P-glycoprotein. Data for Figures 4A and B were also fitted to equation 3 describing the concerted model (see Scheme 1), yielding parameter values for Figure 4A of $K_{iV} = 70.6$ nM, $V_{max} = 0.48$ pmol min⁻¹ mg membrane protein⁻¹ and $L_i = 1.70 \times 10^{-8}$, and for Figure 4B of $K_{iV} = 456$ nM, $V_{max} = 1.70$ pmol min⁻¹ mg membrane protein⁻¹ and $L_i = 0.049$. The simulated curves through the data of Figures 4A and B were generated using these parameter values and equation 3. The increase in the allosteric equilibrium constant, $L_i$, from $1.70 \times 10^{-8}$ to 0.049 in the

© Cancer Research Campaign 1998

Figure 4 Effects of 2-CPZ on the initial uptake rate of VBL by membrane vesicles as a function of extravesicular VBL concentration. Initial uptake rates were determined at the indicated concentrations of [$^3$H]VBL from three time points as described in Materials and methods. **A** Control. **B** plus 5 µM 2-CPZ. Data for both experiments were fitted to the Michaelis–Menten and Hill equations and the equation describing a Concerted Model, as described in Materials and methods (equations 1, 2 and 3) and Results. The parameter values obtained from the Concerted Model were used to draw the lines through the experimental data for **A** and **B**

Figure 5 Effect of 4-CPZ on [$^3$H]VBL accumulation by membrane vesicles. 4-CPZ (10 µM) was added either at the start of the incubation (■) or after the steady state had been reached (▼). Control [$^3$H]VBL accumulation was determined as for Figure 1

presence of 2-CPZ is consistent with an increase in the proportion of a conformer of P-glycoprotein ($P_A$) that does not bind VBL (see Scheme 1). The apparent increase in $V_{max}$ is also of interest and is under further investigation.

### Effect of 4-chlorpromazine on vinblastine accumulation

A different behaviour was observed with 4-CPZ, an analogue of 2-chlorpromazine in which the chlorine on aromatic ring C of the tricyclic structure is at position 4 rather than 2. When 4-CPZ was added at the steady state of [$^3$H]VBL uptake, the accumulated VBL effluxed rapidly but then the intravesicular [$^3$H]VBL concentration returned to the original steady-state level (Figure 5). A second application of 4-CPZ at the new steady state again resulted in rapid VBL efflux (results not shown). When 4-CPZ was added from the start of the incubation, it inhibited VBL transport similarly to 2-CPZ (cf. Figures 5 and 1).

### Effects of colchicine and phenoxybenzamine on vinblastine and 2-chlorpromazine accumulation

We have shown that 2-CPZ is actively transported by these inside-out vesicles (Syed et al, 1996). VBL (10 µM) fully inhibited 2-CPZ accumulation when added at the beginning of the incubation and, when it was added after the steady-state for 2-CPZ accumulation had been reached, it induced efflux of 2-CPZ, which was more rapid than expected from the initial rate of uptake of 2-CPZ (see Syed et al, 1996). Thus, VBL and 2-CPZ have reciprocal effects on the transport of each other. By contrast 10 µM colchicine, a P-glycoprotein substrate in these vesicles (unpublished observations), inhibited VBL uptake by approximately 50% but had no effect on 2-CPZ transport (Table 1). Similarly, 10 µM phenoxybenzamine, a calmodulin antagonist like 2-CPZ, did not inhibit 2-CPZ transport, whereas at this concentration it inhibited VBL transport by approximately 70% (Table 1).

### DISCUSSION

MDR acquired during chemotherapy may in some cases be attributed to overexpression of P-glycoprotein, which actively extrudes anti-cancer drugs from cells. The P-glycoprotein structure consists of two halves, each with six transmembrane domains and one ATP binding site (Azzaria et al, 1989; Rosenberg et al, 1997). There is evidence that hydrolysis of ATP at both sites is required for substrate transport (see Stein, 1997). Most of the compounds transported via P-glycoprotein are lipophilic and cationic at physiological pH (Beck and Qian, 1992). This broad specificity has suggested that lipophilic compounds with low toxicity could be used to saturate and thereby functionally inactivate P-glycoprotein, enabling the

 © Cancer Research Campaign 1998

**Table 1** Inhibitory effects of drugs on vinblastine (VBL) and chlorpromazine (2-CPZ) accumulation by membrane vesicles The effects of 10 μM each of VBL. colchicine or phenoxybenzamine on VBL and 2-CPZ transport were studied. The drugs. dissolved in dimethyl sulphoxide. were added to vesicles in the reaction medium and incubated for 10 min before the addition of 60 nM [³H]VBL or [³H]CPZ to start transport. as described in Materials and methods. The final concentration of dimethyl sulphoxide never exceeded 1% (v/v). Values for per cent inhibition are mean ± s.d. of three experiments

| **Drug** | **Per cent inhibition of accumulation** | |
|---|---|---|
| | **VBL** | **2-CPZ** |
| Vinblastine | 100 | 100 |
| Colchicine | 45.5 ± 2.8 | 0 |
| Phenoxybenzamine | 70.8 ± 8.0 | 5.0 ± 0.9 |

retention of cytotoxic drugs within cells. However. most of the chemosensitizers used so far. although effective in inhibiting P-glycoprotein-mediated transport. have had toxic side-effects at the concentrations needed for the inhibition (Ford et al. 1989). Reversal of drug resistance with reduced in vivo toxicity might be achieved with pharmacologically inactive analogues of the anti-cancer drugs or a combination of two or more chemosensitizing agents. as their ability to reverse drug resistance may be additive or synergistic (Stein. 1997). Reversal of MDR might be more effective clinically if the drugs used acted by different mechanisms or had different binding sites on P-glycoprotein. The present studies were carried out to explore these possibilities in inside-out membrane vesicles prepared from multidrug-resistant human CCRF-CEM leukaemia cells. This vesicle preparation offers a unique system for examining the kinetics of drug transport free of many of the interactions that could affect the kinetics of drug transport in intact cells (see Stein. 1997).

2-CPZ (10 μM) blocked the initial accumulation of [³H]VBL by vesicles almost completely (Figure 1). When 2-CPZ (10 μM) was added at the steady state of [³H]VBL accumulation (after 10–15 min). VBL was effluxed from the vesicles. consistent with inhibition by 2-CPZ of [³H]VBL uptake. However. the rate of efflux of VBL was considerably more rapid than the initial rate of accumulation. being 80% complete within 5 min after 2-CPZ addition. A number of possible mechanisms for this unexpected effect were considered. Phenothiazines are membrane-active agents that can disrupt membrane structure and lead to cell lysis and increased membrane permeability (Saito et al. 1989). However. the rapid [³H]VBL efflux following 2-CPZ addition did not appear to be due to a general increase in membrane permeability. as in a parallel experiment addition of the same concentration of 2-CPZ (10 μM) to vesicles at steady state of $^{45}Ca^{2+}$ accumulation catalysed by the $Ca^{2+}$-pump ATPase did not cause $^{45}Ca^{2+}$ to efflux from the vesicles (Figure 3). This result suggests that under the conditions used the effect of 2-CPZ is selective for the P-glycoprotein-mediated transport process. Similar rapid effluxes were seen when unlabelled VBL was added at the steady state for accumulation of [³H]VBL (unpublished results) or 2-[³H]CPZ (Syed et al. 1996). The pathway for VBL efflux from the inside-out vesicles. equivalent to influx in right side-oriented cancer cells. is not known. The rapid efflux of [³H]VBL was specific to P-glycoprotein ligands (unlabelled VBL or 2-CPZ). as blocking of vinblastine transport by complete inhibition of the ATPase activity of P-glycoprotein with sodium vanadate induced [³H]VBL efflux at a rate corresponding to the initial rate of VBL accumulation (Figure 2). When 2-CPZ was added with vanadate. a rate of efflux intermediate between those observed after addition of 2-CPZ and vanadate singly was observed (Figure 2).

Data for the initial accumulation of VBL or 2-CPZ by P-glycoprotein by inside-out vesicles and the effects of inhibitors of drug accumulation are consistent with the concerted model of Monod et al (1965). in which P-glycoprotein has two binding sites for drugs on the outside of vesicles (consistent with a Hill coefficient of 1.23) and two on the inside. These pairs of sites have a similar affinity for the drug. either both high or low. hence the term 'concerted model'. We propose that P-glycoprotein exists in two conformational states. $P_A$ and $P_B$. 2-CPZ is bound and transported by $P_A$. whereas VBL is not. and $P_B$ binds and transports VBL but not CPZ. The various species of P-glycoprotein present initially are shown in Scheme 1.

Scheme 1

where. for example. $P_A.CC$ and $P_B.VV$ are the two conformers of P-glycoprotein with 2-CPZ and VBL. respectively. bound at two sites on the outer side of the vesicle: $K_{iC}$ and $K_{iV}$ are dissociation constants for the binding of 2-CPZ and VBL. respectively. and $L_i$ is the allosteric equilibrium constant (equation 4).

$$L_i = [P_A]/[P_B] \quad (4)$$

The saturation curve for VBL transport as a function of the external concentration of VBL follows a hyperbolic function (Figure 4A) and it is therefore concluded that in the absence of ligands a significant proportion of P-glycoprotein is the conformer $P_B$. In the presence of 2-CPZ (5 μM). the proportion of P-glycoprotein as $P_A$ is increased and the saturation curve for VBL uptake as a function of VBL concentration becomes sigmoidal (Figure 4B). When the VBL concentration increases. the proportion of $P_B$ capable of mediating VBL uptake increases. generating a sigmoidal saturation curve. The Hill coefficient obtained in the presence of 5 μM 2-CPZ. $n$ = 1.23. is consistent with interaction of VBL with two external sites on P-glycoprotein of inside-out vesicles. This concerted model can be extended to the subsequent steady state where VBL has accumulated in vesicles and 2-CPZ is then added. inducing a very rapid efflux of VBL from the preloaded vesicles (Figure 1). We propose that in preloaded vesicles the two internal binding sites of P-glycoprotein (facing the vesicle lumen) are occupied by VBL at saturating internal concentrations. indicated by the ligands (VV) written to the left of the acceptor. $P_A$:

© Cancer Research Campaign 1998 

$$VV.P_A \underset{K_C}{\rightleftharpoons} VV.P_A.C \underset{K_C}{\rightleftharpoons} VV.P_A.CC$$

$$L \updownarrow$$

$$VV.P_B \underset{K_V}{\rightleftharpoons} VV.P_B.V \underset{K_V}{\rightleftharpoons} VV.P_B.VV$$

Scheme 2

where the saturated P-glycoprotein complex, $VV.P_A.CC$, with VBL bound on the inside and 2-CPZ bound on the outside, enables the very rapid efflux of VBL observed from the steady state when 2-CPZ is added (Figure 1). The converse is also true: there is very rapid efflux of 2-CPZ accumulated in vesicles to a steady state when VBL is then added (see Syed et al, 1996), because of formation of the saturated complex, $CC.P_B.VV$, which rapidly effluxes 2-CPZ.

There are several reasons why P-glycoprotein may exist in two conformational states with specificity for VBL or CPZ. It has been proposed that P-glycoprotein is able to pump out hydrophilic drugs from the internal aqueous cytosol of cells (see Higgins and Gottesman, 1992) and also, lipid-soluble drugs may be pumped out from the lipid bilayer (Rosenberg et al, 1997). The reason for putative differential transport pathways is not known. P-glycoprotein may have one conformational state, $P_A$, which binds and pumps classes of drugs such as 2-CPZ from one site in the lipid bilayer into vesicles and a second conformational state, $P_B$, which binds and pumps drugs such as VBL from a different lipid phase into vesicles. All the saturated complexes of P-glycoprotein ($VV.P_A.CC$, $CC.P_B.VV$ and $VV.P_B.VV$) would rapidly efflux the internal ligand of the inside-out vesicles (on the left) to the exterior. We have found that verapamil has similar inhibitory effects on VBL uptake to 2-CPZ: it also induces the rapid efflux of VBL from preloaded vesicles (cf. Figure 1) and sigmoidality in the saturation curve for VBL uptake as a function of VBL concentration (cf. Figure 4B; data not shown).

The data of Table 1 provide further evidence that P-glycoprotein may exist in two conformational states. Colchicine and phenoxybenzamine are effective inhibitors of VBL uptake by vesicles, but at the same concentrations have little effect on the uptake of 2-CPZ. There is evidence for distinct sites on P-glycoprotein for substrates and chemosensitizers. Whereas vincristine, VBL, verapamil and reserpine effectively inhibit binding of a photoaffinity analogue of VBL and azidopine to P-glycoprotein, inhibition by the phenothiazines, 2-CPZ and trifluoperazine, and by chloroquine, is poor (Safa et al, 1986; Akiyama et al, 1988).

The concerted, two-state model of Schemes 1 and 2 is also consistent with the inhibition of VBL accumulation by vanadate (Figure 2). Addition of vanadate to vesicles that had accumulated VBL to the steady-state level resulted in efflux of VBL at a similar rate to the initial accumulation rate as a result of inhibition of the ATPase activity of P-glycoprotein (Figure 2). We conclude that the saturated complexes of P-glycoprotein can only rapidly efflux the internal ligand with concurrent ATPase activity. The finding that P-glycoprotein acts efficiently as a drug -efflux pump with low but not high concentrations of anti-cancer agents outside target cells (Miyamoto et al, 1996) might be accounted for by rapid influx of the anti-cancer agents through the pump, consistent with data presented here, or by inhibition of the efflux pathway in intact cells when external sites are saturated.

The model can also account for the transitory inhibition of VBL accumulation induced by addition of 4-CPZ at steady state (Figure 5). When 4-CPZ is added at the steady state for VBL transport, the complex $VV.P_A.CC$ would be formed, with consequent rapid efflux of VBL (Figure 5). However, with the loss of VBL from inside the vesicle upon its rapid efflux, the complex $P_A.CC$ would predominate and if the dissociation constant $K_C$ were higher for 4-CPZ than for 2-CPZ, VBL could bind to form $P_B.VV$, with dissociation of the 4-CPZ. At this stage, the transport of VBL would resume, as shown in Figure 5. This effect should be reversed on a second addition of 4-CPZ, as was indeed found to be the case. Ford et al (1989) have shown the importance of substitution at the 2-position of ring C of the phenothiazines for anti-MDR activity and demonstrated that 2-CPZ has a higher MDR ratio than 4-CPZ. The significance of this oscillatory behaviour of the P-glycoprotein pump remains to be determined.

The minimal concerted two-state model summarized in Schemes 1 and 2 is consistent with the data presented in this paper. Equation 3 derived from the concerted model proposed here for P-glycoprotein (Schemes 1 and 2) was able to satisfactorily simulate the data shown for VBL uptake in the absence or presence of 2-CPZ (Figures 4A and B), but in the absence of 2-CPZ the data was well approximated by a simple hyperbolic function (equation 1, Fig. 4A). We propose to further test and refine this model using a continuous, real-time assay rather than the stopped-time radioassay used here. This model provides an alternative explanation for the mechanism of action of reversal agents that sensitize multidrug-resistant cancer cells to anti-cancer drugs. A non-toxic reversal agent could be administered first to the patient to bind to internal sites in cells of the right side-out P-glycoprotein. A cytotoxic drug such as vinblastine subsequently administered could then rapidly enter multidrug-resistant cells with high levels of P-glycoprotein via saturated complexes formed because of internal binding of the reversal agent. Thus, all cells expressing high levels of P-glycoprotein would be selectively exposed to the subsequently administered anti-cancer drug.

### ABBREVIATIONS

MDR, multidrug resistance; VBL, vinblastine; 2-CPZ, 2-chlorpromazine (2-chloro-10-(3-dimethylaminopropyl)-phenothiazine); 4-CPZ, 4-chlorpromazine (4-chloro-10-(3-dimethylaminopropyl)-phenothiazine).

### ACKNOWLEDGEMENTS

The authors wish to thank Dr Ross Davey, Royal North Shore Hospital, Sydney, NSW, for providing the VBL-resistant CCRF-CEM cell line. Valuable discussions with Drs David Cutler and Michael Morris on kinetic aspects and assistance of Dr Kai Ling Wang with curve-fitting procedures were greatly appreciated. We thank Mary Bebawy for assistance with the preparation of the manuscript. Financial support from the University of Sydney Cancer Research Fund, NSW State Cancer Council and Leo & Jenny Leukaemia and Cancer Foundation is acknowledged.

 © Cancer Research Campaign 1998

### REFERENCES

Akiyama S-I. Cornwell MM. Kuwano M. Pastan I and Gottesman MM (1988) Most drugs that reverse multidrug resistance also inhibit photoaffinity labelling of P-glycoprotein by a vinblastine analog. *Mol Pharmacol* **33**: 144–147

Arias I (1990) Multidrug resistance genes. P-glycoprotein and the liver. *Hepatology* **12**: 159–165

Azzaria ME. Schurr E and Gros P (1989) Discrete mutations introduced in the predicted nucleotide-binding sites of the mdr1 gene abolish its ability to confer multidrug resistance. *Mol Cell Biol* **9**: 5289–5287

Beck WT and Qian X-D (1992) Photoaffinity substrates for P-glycoprotein. *Biochem Pharmacol* **43**: 89–93

Ferry DR. Russell MA and Cullen MH (1992) P-glycoprotein possesses a 1.4-dihydropyridine-selective drug acceptor site which is allosterically coupled to a vinca-alkaloid-selective binding site. *Biochem Biophys Res Commun* **188**: 440–445

Fojo A. Akiyama SI. Gottesman MM and Pastan I (1985) Reduced drug accumulation in multiple drug-resistant human KB carcinoma cell lines. *Cancer Res* **45**: 3002–3007

Ford JM. Prozialeck WC and Hait WN (1989) Structural features determining activity of phenothiazines and related drugs for inhibition of cell growth and reversal of multidrug resistance. *Mol Pharmacol* **35**: 105–115

Gottesman MM and Pastan I (1988) The multidrug transporter. a double-edged sword. *J Biol Chem* **263**: 12163–12166

Hamada H and Tsuruo T (1988) Purification of the 170- to 180-kilodalton membrane glycoprotein associated with multidrug resistance. 170- to 180- kilodalton membrane glycoprotein is an ATPase. *J Biol Chem* **263**: 1454–1458

Higgins CF and Gottesman MM (1992) Is the multidrug transporter a flippase? *Trends Biochem Sci* **17**: 18–21

Hofsli E and Nissen-Meyer J (1990) Reversal of multidrug resistance by lipophilic drugs. *Cancer Res* **50**: 3997–4002

Horio M. Gottesman MM and Pastan I (1988) ATP-dependent transport of vinblastine in vesicles from human multidrug-resistant cells. *Proc Natl Acad Sci USA* **88**: 3580–3584

Malkhandi J. Ferry DR. Boer R. Gekeler V. Ise W and Kerr DJ (1994) Dexniguldipine-HCl is a potent allosteric inhibitor of [$^3$H]vinblastine binding to P-glycoprotein of CCRF ADR 5000 cells. *Eur J Pharmacol* **288**: 105–114

McLaughlin S and Eisenberg M (1975) Antibiotics and membrane biology. *Annu Rev Biophys Bioeng* **4**: 335–366

May GL. Wright LC. Dyne M. Mackinnon WB. Fox RM and Mountford CE (1988) Plasma membrane lipid composition of vinblastine sensitive and resistant human leukaemic lymphoblasts. *Int J Cancer* **42**: 728–733

Miyamoto KI. Koga-Takeda K. Koga K. Ohshima T and Nomura M (1996) Saturable function of P-glycoprotein as a drug-efflux pump in multidrug-resistant tumour cells. *J Pharm Pharmacol* **48**: 522–525

Monod J. Wyman J and Changeux JP (1965) On the nature of allosteric transitions: a plausible model. *J Mol Biol* **12**: 88–95

Nooter K. Oostrum R. Jonker R. Van Dekken H. Stokdijk W and Van Den Engh G (1989) Effect of cyclosporin A on daunorubicin accumulation in multidrug resistant P388 leukaemia cells measured by real-time flow cytometry. *Cancer Chemother Pharmacol* **23**: 296–300

Pereira E. Borrel MN. Fiallo M and Garnier-Suillerot A (1994) Non-competitive inhibition of P-glycoprotein-associated efflux of THP-adriamycin by verapamil in living K562 leukemia cells. *Biochim Biophys Acta* **1225**: 209–216

Rosenberg MF. Callaghan R. Ford RC. Higgens CF (1997) Structure of the multidrug resistance P-glycoprotein to 2.5 nm resolution determined by electron microscopy and image analysis. *J Biol Chem* **272**: 10685–10694

Safa AR. Glover CJ. Meyers M. Biedler JL and Felsted RL (1986) Vinblastine photoaffinity labelling and a high molecular weight surface membrane glycoprotein specific for multidrug-resistant cells. *J Biol Chem* **261**: 6137–6140

Saito H. Kawai S. Iseki K. Miyazaki K and Arita TJ (1989) Transport characteristics of [$^3$H]-chlorpromazine across rat small intestinal brush border membrane. *J Pharm Pharmacol* **41**: 200–202

Sehested M. Skovsgaard P. Jensen PB. Demant EJ. Friche E and Bindslev N (1990) Transport of the multidrug resistance modulators verapamil and azidopine in wild type and daunorubicin resistant Ehrlich ascites tumour cells. *Br J Cancer* **62**: 37–41

Shibata H and Ghishan FK (1990) Intestinal calcium transport in spontaneously hypertensive rats (SHR) and their genetically matched WK4 rats. *Proc Soc Exp Biol Med* **194**: 26–31

Spoelstra EC. Westerhoff HV. Dekker H. Lankelma J (1992) Kinetics or daunorubicin transport by P-glycoprotein of intact cancer cells. *Eur J Biochem* **207**: 567–579

Stein WD (1997) Kinetics of the multidrug transporter (P-glycoprotein) and its reversal. *Physiol Rev* **77**: 545–590

Syed SK. Christopherson RI and Roufogalis BD (1993) Vinblastine transport by membrane vesicles from human multi-drug resistant CCRF-CEM leukaemia cells: inhibition by taxol and membrane permeabilising agents. *Biochem Mol Biol Int* **30**: 743–753

Syed SK. Christopherson RI and Roufogalis BD (1996) Chlorpromazine transport in membrane vesicles from multidrug resistant CCRF-CEM cells. *Biochem Mol Biol Int* **39**: 687–696

Tamai I and Safa AR (1991) Azidopine non-competitively interacts with vinblastine and cyclosporin A binding to P-glycoprotein in multidrug resistant cells. *J Biol Chem* **266**: 16796–16800

Tsuruo T. Lida H. Tsukagoshi S and Sakurai Y (1982) Increased accumulation of vincristine and adriamycin in drug-resistant P388 tumour cells following incubation with calcium antagonists and calmodulin inhibitors. *Cancer Res* **42**: 4730–4733

© Cancer Research Campaign 1998